# TNFα-YAP/p65-HK2 axis mediates breast cancer cell migration

**DOI:** 10.1038/oncsis.2017.83

**Published:** 2017-09-25

**Authors:** Y Gao, Y Yang, F Yuan, J Huang, W Xu, B Mao, Z Yuan, W Bi

**Affiliations:** 1 State Key Laboratory of Brain and Cognitive Sciences, Institute of Biophysics, Chinese Academy of Sciences, Beijing, China; 2 College of Life Sciences, University of Chinese Academy of Sciences, Beijing, China; 3 The Brain Science Center, Beijing Institute of Basic Medical Sciences, Beijing, China; 4 Department of Oncology, Chinese PLA General Hospital, Beijing, China; 5 Department of Orthopedics, Clinical Division of Surgery, Chinese PLA General Hospital, Beijing, China; 6 General Surgery Center, Chinese PLA General Hospital, Beijing, China; 7 Center of Alzheimer's Disease, Beijing Institute for Brain Disorders, Beijing, China

## Abstract

Clinical and experimental evidence indicates that macrophages could promote solid-tumor progression and metastasis. However, the mechanisms underlying this process remain unclear. Here we show that yes-associated protein 1 (YAP1), a transcriptional regulator that controls tissue growth and regeneration, has an important role in tumor necrosis factor α (TNF α)-induced breast cancer migration. Mechanistically, macrophage conditioned medium (CM) or TNFα triggers IκB kinases (IKKs)-mediated YAP phosphorylation and activation in breast cancer cells. We further found that TNFα or macrophage CM treatment increases the interaction between p65 and YAP. Chromatin immunoprecipitation (ChIP) assay shows that YAP/TEAD (TEA domain family member) and p65 proteins synergistically regulate the transcription of hexokinase 2 (HK2), a speed-limiting enzyme in glycolysis, and promotes TNFα-induced or macrophage CM-induced cell migration. Together, our findings indicate an important role of TNFα-IKK-YAP/p65-HK2 signaling axis in the process of inflammation-driven migration in breast cancer cells, which reveals a new molecular link between inflammation and breast cancer metastasis.

## Introduction

Tumor microenvironment consists of heterogeneous components including extracellular matrix, tumor-associated stromal cells and a myriad of signaling molecules,^1^ which can significantly influence tumor growth and metastasis.^2^ Macrophages in tumor microenvironment have a key role in promoting tumor metastasis.^3^

TNFα, mainly derived from activated macrophages, is a well-known cytokine that regulates the inflammatory processes in tumor development. High level of tumor necrosis factor α (TNF α) is associated with an aggressive behavior and a poor prognosis in many malignant cancers, including breast cancers.^4^ Studies reported that TNFα induces epithelial–mesenchymal transition and further facilitates metastasis in breast cancer and prostate cancer.^5^ The signaling mechanisms underlying the pro-invasive activity of TNFα are still largely unknown. In tumor cells, TNFα activates IκB kinases (IKKs), c-Jun N-terminal kinase and mitogen-activated protein kinase signaling to stimulate the nuclear translocation of transcription factors including activator protein-1 (AP-1) and nuclear factor kappa B (NF-κB) via TNF receptor 1 (TNFR1).^6^ TNFα promotes the expression of genes involved in tumor invasion and metastasis such as interleukin-8 (IL-8), monocyte chemotactic protein-1 and matrix metalloproteinase, thus enhancing tumor progression.^6, 7^

The Hippo pathway is a highly conserved signaling that controls organ size and is tightly involved in tumorigenesis. The core components of the Hippo pathway constitute a kinase cascade. In complex with Sav1, Mst1/2 phosphorylates and activates Lats1/2. Lats1/2 phosphorylates yes-associated protein (YAP)/TAZ and promotes the binding of YAP/TAZ to 14−3−3, which leads to cytoplasmic retention of YAP/TAZ. YAP/TAZ, in conjunction with TEA domain family members (TEAD1–4), mediates the major physiological functions of the Hippo pathway.^8, 9^ The roles of YAP in oncogenesis, including the promotion of cell proliferation, the inhibition of apoptosis and the induction of the epithelial–mesenchymal transition, have been elucidated.^9, 10, 11, 12^ Many upstream signaling contributes to tumorigenesis have been found to activate YAP. For example, hypoxia stimulates YAP though SIAH2-mediated degradation of LATS2.^13^ Recently, it was reported that intestinal IL-6-gp130 signaling triggers activation of YAP that dependent on Src-mediated phosphorylation to maintain epithelial cell proliferation, providing the evidence that YAP is responsive to the inflammatory microenvironment.^14^ However, whether YAP also has an essential role in inflammation-associated tumor progression is still largely unknown.

In our study, we found that TNFα triggers IKK-mediated YAP phosphorylation and activation in breast cancer cells. We found that conditioned medium (CM) from macrophage or TNFα treatment stabilizes YAP protein and increases the interaction between YAP and p65. Further, YAP/TEAD/p65 triplet synergistically upregulates hexokinase 2 (HK2) transcription, which promotes breast cancer cell migration. Thus, our results uncovered a non-autonomously regulatory mechanism of YAP in cancer cells by environmental cues and provided a molecular basis for targeting TNFα-IKK-YAP/p65-HK2 pathway to effectively treat breast cancer cell metastasis.

## Results

### Macrophage CM treatment promotes the transactivation of YAP

**Figure 1:**
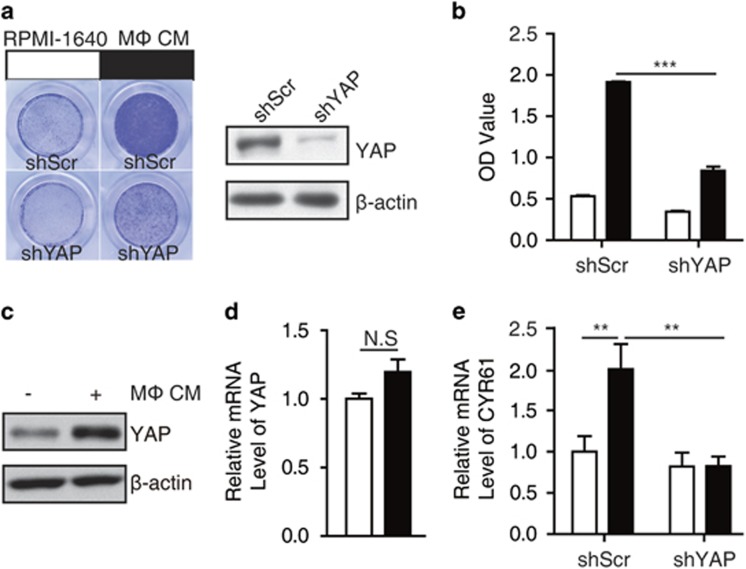
Macrophage CM promotes cell migration and YAP activation. (**a**) Control or shYAP stably transfected MCF7 cells were cultured with control medium or macrophage CM in the transwells for 24 h. The migratory ability was determined by transwell assay. The right panel showed the YAP knockdown efficiency. (**b**) The transwell membranes were washed by dimethylsulfoxide (DMSO) and the OD values were calculated at the absorbance of 570 nm. Data were collected from three independent experiments. (**c**, **d**) MCF7 cells were cultured in control medium or macrophage CM for 24 h, YAP expression was detected by western blot (**c**) and real-time PCR (**d**). (**e**) Control or shYAP stably transfected MCF7 cell lines were cultured with macrophage CM for 24 h and then the CYR61 mRNA level was assessed *via* real-time PCR. The error bars represent the means±s.d. (NS, no significance; **P<0.01; ***P<0.001, *n*=3).

YAP is overexpressed in various cancers and closely related to breast cancer tumorigenesis.^15, 16, 17, 18, 19, 20, 21^ YAP could promote cancer cell migration, and we hypothesized that YAP might be involved in macrophage-mediated and inflammation-induced cancer cell metastasis. First, we established MCF7 breast cancer cells stably expressing YAP short hairpin RNAs (shRNA) via lentiviral infection. Then, the stable cell lines were exposed to CM from cultured human THP-1 macrophages. The ability of cell migration was measured by transwell assay. The results showed that macrophage CM significantly increased the migration of MCF7 cells, whereas knockdown of YAP rescued this phenomenon (Figures 1a and b). This evidence prompted us to investigate whether macrophage CM stimulated the activity of YAP. As expected, we found the protein level of YAP increased upon macrophage CM treatment (Figure 1c) and the mRNA level of YAP is not changed (Figure 1d).

To determine whether the increase of YAP levels was associated with its functional activation, we examined the expression of YAP target gene CYR61 in MCF7 cells. As expected, macrophage CM increased the expression of CYR61 and this effect was attenuated by YAP knockdown, indicating the activity of YAP is enhanced by macrophage-secreted factors (Figure 1e). Taken together, these results argue that macrophage-mediated inflammation increased YAP protein levels and transcriptional activity in breast cancer cells.

### TNFα controls YAP activity in breast cancer cells

**Figure 2:**
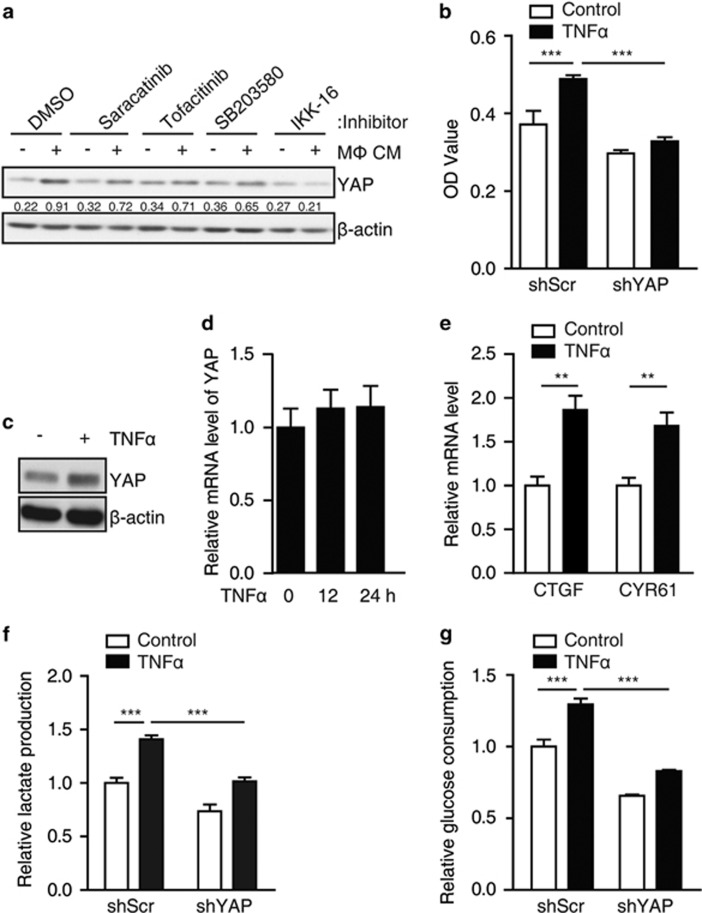
TNFα increases the activation of YAP in breast cancer cells. (**a**) MCF7 cells were cultured with control medium or macrophage CM, and simultaneously treated with 0.5 μM Src-inhibitor saracatinib, 10 μM JAK-inhibitor tofacitinib, 10 μM mitogen-activated protein kinase-inhibitor SB203580 or 10 μM IKK-inhibitor IKK-16 for 24 h, the cells were lysed and YAP protein level was monitered by western blot. (**b**) Control or shYAP MCF7 cell lines were treated with 10 μM TNFα for 24 h and the cell migration was demonstrated by transwell assay, the results were shown as the invasive cell OD value. Data were collected from at least three independent experiments. (**c**) MCF7 cells were treated with 10 μM TNFα for 24 h, the cell lysates were subject to western blot to detect YAP expression. (**d**) MCF7 cells were treated with 10 μM TNFα for 12 h, YAP mRNA level was measured by real-time PCR. (**e**) MCF7 cells were treated with 10 μM TNFα for 12 h and the mRNA levels of CTGF and CYR61 were measured by real-time PCR. (**f**, **g**) Control and shYAP MCF7 cells were treated with 10 μM TNFα for 12 h. Lactate production (**f**) and glucose consumption (**g**) were measured. The error bars represent the means±s.d. (**P<0.01; ***P<0.001; *n*=3).

Inflammatory cytokines secreted by macrophages, such as TNFα, IL-1 and IL-6, have been reported to take part in tumor cell malignancy.^22, 23, 24^ These cytokines promote the metastasis of tumor cells via the activation of different signaling pathways. Then, we used the specific inhibitors of these cytokines to define which inflammatory signaling is involved in macrophage-stimulated YAP protein stability and activation. Among the inhibitors used, only IKK-16, which inhibits the activity of IKKs, could significantly reduce macrophage CM-induced YAP protein upregulation (Figure 2a). As TNFα is the major upstream cytokine for IKK activation, we chose TNFα as the cytokine that activates YAP signaling. First, we found that TNFα treatment increased MCF7 cell migration in transwell assay and YAP knockdown reduced TNFα-induced breast cancer cell migration (Figure 2b). Second, we observed that TNFα treatment upregulated YAP protein level (Figure 2c), but not mRNA level (Figure 2d). In addition, we found that TNFα induced an increased nuclear localization of YAP but independent on Ser127 phosphorylation (Supplementary Figures 1a and b). Finally, TNFα treatment also increased the mRNA levels of the tested target genes of YAP (CYR61 and CTGF) in MCF7 cells (Figure 2e). Together, similar to macrophage CM, TNFα treatment could activate YAP signaling in breast cancer cells.

### YAP mediates TNFα-induced expression of HK2 and glycolysis in breast cancer cells.

**Figure 3:**
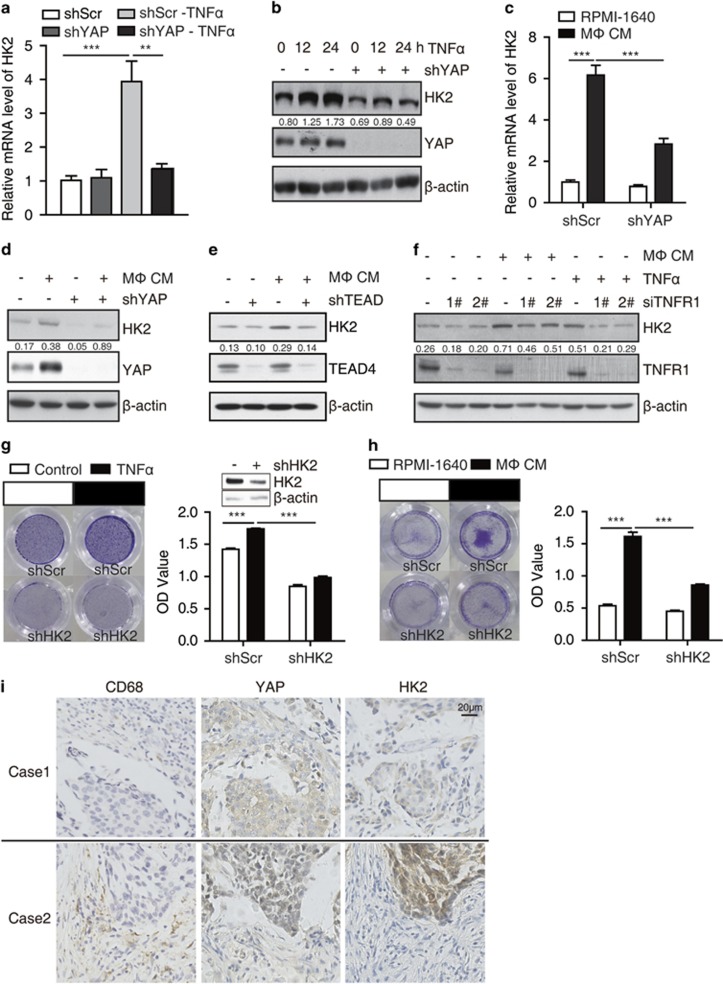
YAP mediates TNFα-induced expression of HK2 and cell migration in breast cancer cells. (**a**, **b**) Control and shYAP MCF7 cells were treated with 10 μM TNFα for 12 h or 24 h. HK2 mRNA and protein levels were detected *via* real-time PCR (**a**) and western blot (**b**). (**c**, **d**) Control and shYAP MCF7 cells were cultured with control medium or the indicated macrophage CM for 12 h. HK2 mRNA and protein levels were determined by real-time PCR (**c**) and western blot (**d**). (**e**) Control and shTEAD MCF7 cells were cultured in control medium or macrophage CM. The protein level of HK2 was determined by western blot. (**f**) MCF7 cells were transfected with siRNA for TNFR1 and then cultured in macrophage CM or treated with 10 μM TNFα. The cells were lysed for the detection of HK2 expression. (**g**, **h**) MCF7 cells stably expressing shRNA targeting HK2 were cultured in macrophage CM (**g**) or treated with 10 μM TNFα (**h**) in transwells for 24 h. Cell migration were analyzed by transwell assay. (**i**) Clinical specimens of breast cancer were immunostained with antibodies against CD68, YAP and HK2, the typical specimens of the same region were exhibited, the classification and the correlation between two proteins were performed. The error bars represent the means±s.d. (**P<0.01; ***P<0.001; *n*=3).

Interestingly, we found that the culture medium of TNFα-treated MCF7 cells turned yellow much faster than control cells, whereas YAP knockdown blunted this phenotype. Then, we hypothesized that this phenomenon might be because of lactate acidosis, the byproduct of glycolysis. As expected, we observed that there were significantly less lactate production and glucose consumption in the YAP knockdown cells compared with the control (Supplementary Figures 2a and b). To explore the cellular mechanism that YAP regulates glucose metabolism, we examined the mRNA levels of HK2, LDHs, PKM2, PFKL and GLUT4, which encode the key enzymes responsible for glycolysis and have been reported as putative targets of YAP signaling. The real-time PCR (RT–PCR) assay showed that only HK2 was significantly downregulated in YAP knockdown cells (Supplementary Figure 2c). In addition, we found that either YAP knockdown (Supplementary Figures 2d and e) or TEAD1/3/4 knockdown (Supplementary Figures 2f and g) markedly decreased the mRNA level of HK2. Similarly, direct TNFα treatment elevated the lactate production and glucose consumption, which was attenuated by YAP knockdown (Figures 2f and g). Further, the expression of HK2 could be upregulated by TNFα treatment in MCF7 cells and YAP knockdown significantly impaired TNFα-induced HK2 expression both at mRNA (Figure 3a) and protein levels (Figure 3b). Interestingly, IL-6 treatment failed to induce HK2 expression in MCF7 cells (Supplementary Figure 2h). In addition, macrophage CM also increased HK2 expression, which could be blocked by knockdown of YAP (Figures 3c and d), TEAD1/3/4 (Figure 3e) or TNFR1 (Figure 3f), indicating that TNFα might be the major component of CM for YAP signaling activation from the cultured macrophage cells. Respectively, TNFα/macrophage CM-promoted cell migration was also inhibited by HK2 knockdown in MCF7 cells (Figures 3g and h). Collectively, our data showed that macrophage-associated inflammation promotes cancer cell migration through YAP-HK2 axis. To further validate that the macrophoages/TNFα could activate YAP and HK2 *in vivo*, we evaluated the relevance of CD68+ (highly expressed in blood monocytes and tissue macrophages) staining with YAP or HK2 staining in human breast cancer samples via immunohistochemical staining. Notably, both YAP and HK2 expression were positively correlated with CD68+ stainng in human breast cancer samples (Figure 3i, Spearman’s correlation, *r* (HK2 vs CD68+)=0.476, *P*<0.01; *r* (YAP vs CD68+)=0.526, *P*<0.01). In addtion, the expression of HK2 also positively correlated with YAP in human breast cancer samples (Spearman’s correlation, *r* =0.581, *P*<0.001), indicating that YAP-mediated HK2 upregulation might exist *in vivo*.

### IKKβ and IKKε phosphorylate YAP

**Figure 4:**
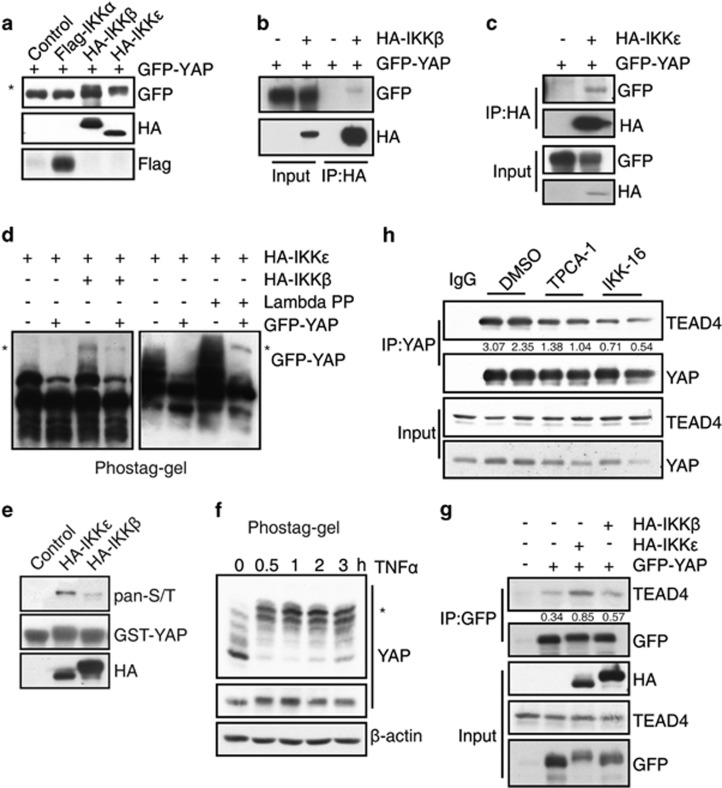
IKKβ and IKKε phosphorylate YAP. (**a**) 293T cells were co-transfected with GFP-YAP and Flag-IKKα, HA-IKKβ or IKKε. The cell lysates were analyzed by western blot with indicated antibodies. The asterisk showed the shifted band (**b**, **c**) 293T cells were co-transfected with GFP-YAP and HA-IKKβ or IKKε. Cell lysates were immunoprecipitated with indicated antibodies and analyzed by western blot. (**d**) 293T cells were transfected with GFP-YAP and HA-IKKβ or IKKε. The cell lysates were analyzed by phos-tag gel western blot. The asterisk showed the phosphorylated band. (**e**) The recombinant GST-YAP protein was incubated with immunoprecipitated HA-IKKβ or IKKε in phosphorylation buffer. Reactions were subjected to electrophoresis and immunoblotted with antibody against pan-phosphorylated serine/threonine (S/T). (**f**) MCF7 cells were treated with TNFα for indicated times, the cells were then lysed for phos-tag gel western blot. The asterisk showed the phosphorylated band. (**g**) 293T cells were transfected with GFP-YAP and HA-IKKβ/ε. Cell lysates were immunoprecipitated with GFP antibody followed by immunoblotting with TEAD4 antibody. (**h**) MCF7 cells were treated with 10 μM TPCA-1 or 10 μM IKK-16 for 12 h. Then endogenous YAP was immunoprecipitated and followed by immunoblotting with TEAD4 antibody.

When cells are treated by TNFα, the intracellular IKK kinase complex consisting of IKKα, IKKβ and NEMO (IKKγ) is activated both in the cytoplasm and nucleus.^25, 26^ It has also been reported that TNFα could induce the nuclear translocation of non-canonical IKKε and lead to its activation.^27^ Similarly, in MCF7 cells TNFα could activate both IKKα/β and IKKε (Supplementary Figure 3a). To assess whether IKKs are responsible for TNFα-mediated YAP regulation, we co-expressed YAP with IKKα, IKKβ or IKKε in 293T cells. Interestingly, we found that overexpression of IKKβ or IKKε led to significant band shift of YAP (Figure 4a). The subsequent co-IP assay showed that YAP physically interacted with IKKβ (Figure 4b) and IKKε (Figure 4c). By using the phos-tag gel filtration, we observed that both IKKβ and IKKε induced a mobility shift of YAP protein, indicating that IKKβ and IKKε mediate YAP phosphorylation in cells (Figure 4d). *In vitro* kinase assay followed by western blot analysis using antibody against pan-phosphorylated serine/threonine further confirmed that IKKβ and IKKε were able to phosphorylate the purified YAP protein (Figure 4e). Consistently, treatment of MCF7 cells with 10 μM TNFα induced rapid and robust YAP phosphorylation (Figure 4f).

As a transcriptional co-factor, YAP needs to bind its transcription factor, TEAD family proteins, to transactivate the downstream target genes. In order to determine if IKK-mediated YAP phosphorylation regulates the interaction between YAP and TEADs, we performed the co-IP assay. We found that YAP–TEAD4 interaction was indeed enhanced by the overexpression of IKKβ or IKKε (Figure 4g). Consistently, TPCA-1 and IKK-16, the inhibitor of IKKs, markedly reduced the endogenous interaction between YAP and TEAD4 (Figure 4h). Similar results were observed when MCF7 cells were transfected with small interfering RNA (siRNA) against IKKβ or IKKε or both (Supplementary Figure 3b). Moreover, inhibition of IKKs by inhibitors or the global siRNAs abrogated the upregulation of HK2 mRNA and protein expression induced by TNFα (Supplementary Figures 3c-e). Altogether, we identified IKKβ/ε as the novel upstream modulators for YAP phosphorylation and revealed a new regulatory mechanism of YAP activation.

### YAP-TEAD and p65 synergistically regulate the expression of HK2

**Figure 5:**
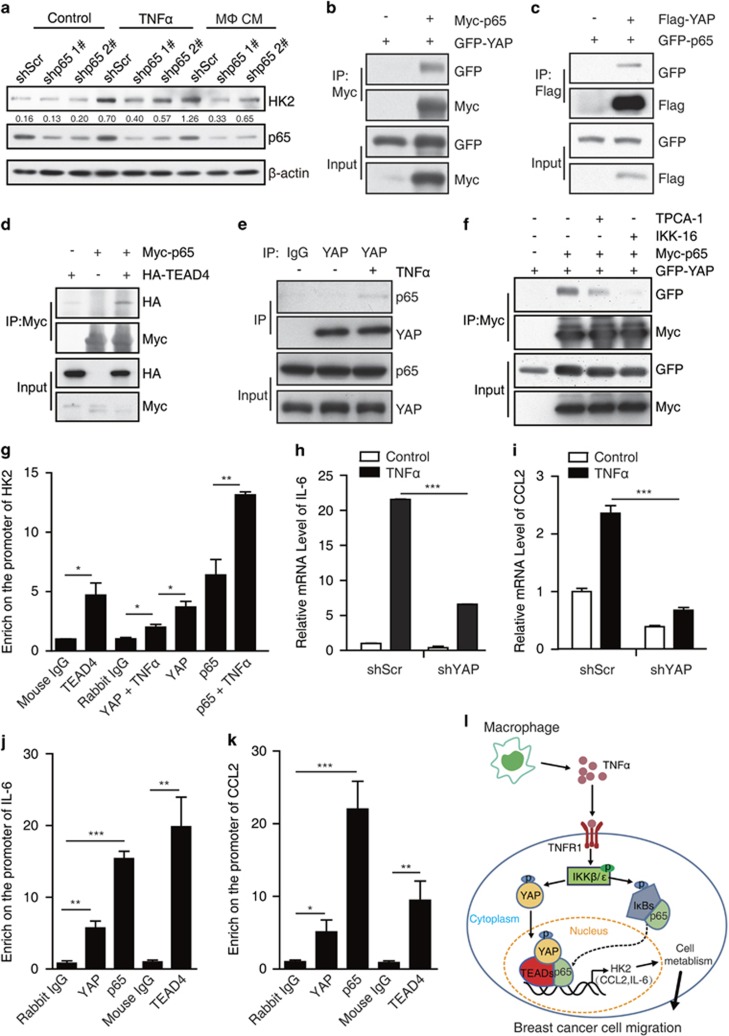
YAP-TEAD and p65 synergistically regulate the expression of HK2. (**a**) MCF7 cell lines stably expressing shRNA (1# and 2#) targeting p65 were treated with control or 10 μM TNFα or macrophage CM for 24 h, p65 knockdown efficiency and HK2 protein level were confirmed by western blot. (**b**) 293T cells were co-transfected with GFP-YAP and Myc-p65. Cell lysates were immunoprecipitated with Myc antibody and then analyzed by western blot. (**c**) 293T cells were co-transfected with GFP-p65 and Flag-YAP. Cell lysates were immunoprecipitated with Flag antibody and then analyzed by western blot. (**d**) 293T cells were transfected with Myc-p65 and HA-TEAD4. Immunoprecipitation of Myc-p65 and co-immunoprecipitation of HA-TEAD4 were detected by western blot. (**e**) MCF7 cells were treated with 10 μM TNFα for 12 h, cell lysates were immunopreipitated with rabbit IgG or YAP antibody, the endogous co- immunopreipitated p65 was measured by immunoblotting. (**f**) 293T cells were co-transfected with GFP-YAP and Myc-p65. After transfection, the cells were treated with 10 μM TPCA-1 or 10 μM IKK-16 for followed 18 h, Myc-p65 was immunoprecipitated with Myc antibody and the co-immunoprecipitated GFP-YAP was determined by western blot. (**g**) MCF7 cells were treated with 10 μM TNFα for 12 h. The cell lysates were used for ChIP analysis with antibody against TEAD4, YAP or p65. The binding of TEAD4, YAP or p65 on HK2 promoter were detected by real-time PCR. (**h**, **i**) Control and shYAP MCF7 cells were treated with TNFα for 12 h. The mRNA levels of CCL2 (**h**) and IL-6 (**i**) were analyzed by real-time PCR. (**j**, **k**) MCF7 cells were treated with 10 μM TNFα for 12 h. Then, the cell lysates were used for ChIP analysis with antibody against TEAD4, YAP or p65. The binding of TEAD4, YAP or p65 on CCL2 or IL-6 promoter were detected by real-time PCR. (**l**) The model of YAP/TEAD/p65 interact with each other to synergistically regulate gene transcription and breast cancer cell migration under TNFα treatment. The error bars represent the means±s.d. (*P<0.05; **P<0.01; ***P<0.001, *n*=3).

It has been reported that inhibition of NF-κB by overexpressing IκBα-supper repressor (IκBα-SR that completely blocks NF-κB signaling) markedly inhibited TNFα-induced HK2 expression in skeletal muscle cells.^28^ Here, we found that NF-κB inhibition or YAP knockdown could block TNFα-induced HK2 expression, we wondered whether there is a crosstalk between YAP and NF-κB pathway on the transcriptional regulation of HK2 in breast cancer cells. The inhibitors of p65 (JSH-23 and PDTC) (Supplementary Figure 3f) or p65 knockdown (Figure 5a) decreased TNFα-induced HK2 upregulation, suggesting that p65 and YAP probably share the same pathway to regulate the expression of HK2. TEAD1/p65 complex has been reported to regulate gene transcription in the innate immune response.^29^ We then examined the physical interaction between YAP/TEAD and p65. Indeed, overexpressed YAP or TEAD4 interacted with p65 in 293T cells (Figures 5b–d) and the endogenous interaction between YAP and p65 was evident under TNFα treatment (Figure 5e). Consistently, the inhibitors of IKKs (TPCA-1 and IKK-16) significantly disrupted the interaction between YAP and p65 (Figure 5f).

To further determine the co-operation of YAP/p65 on the HK2 transcriptional regulation, we performed chromatin Immunoprecipitation (ChIP) assay to investigate whether YAP/TEAD and p65 bind to the same region on the HK2 promoter. We designed the primers for ChIP assay according to p65 ChIP-seq result (UCSC accession: wgencodeEH000735), which contains the region of predicted p65 binding site on HK2 promoter (chr2:75062167–75062536, hg19/Human). As expected, YAP, TEAD4 and p65 all bind to the dedicated region and TNFα treatment increased the enrichment of YAP or p65 on HK2 promoter (Figure 5g).

To further define the synergy and generality of YAP/p65 complex in the regulation of gene transcription upon TNFα treatment, we sought to examine whether the putative p65 targets could be regulated by YAP/TEAD. Similar to HK2 expression, IL-6 or CCL2 upregulation induced by TNFα treatment was impaired by YAP knockdown in MCF7 cells (Figure 5h and i). Further ChIP assay demonstrated that YAP or TEAD4 could bind to the identified p65 associated region of IL-6/CCL2 promoter in response to TNFα treatment (Figures 5j and k). Taken together, YAP/TEAD/p65 interact with each other to synergistically regulate gene transcription (Figure 5l).

## Discussion

The association between inflammation and cancer has been well appreciated in many types of cancer, and the inflammation has been regarded as the ‘seventh hallmark of cancer’.^30^ YAP, as a putative proto-oncogene, has recently been revealed to crosstalk with inflammation signaling during tumor initiation. For example, Kim *et al.*^31, 32^ showed that serum response factor-YAP-IL-6 signaling axis is critical for stemness maintenance of mammary stem cells in basal-like breast cancer. Adenomatous polyposis coli loss results in the Src family kinase-mediated YAP phosphorylation and activation, which lead to upregulation of the IL-6 signal transducer (IL-6ST/gp130) and tumorigenesis of human colorectal cancer.^33^ At present, we demonstrate that YAP functions as a sensor for TNFα, and in turn activated YAP and NF-κB synergistically regulate the downstream gene transcription.

TNFα stimulates both canonical IKKα/β-Nemo complex and non-canonical IKKε, thereby leading to the activation of NF-κB and the expression of downstream genes.^27^ Although degradation of IκBα and activation of NF-κB are believed to be the major cellular function of the IKK complex, there is growing evidence that IKKs have other substrates in inflammatory factors-mediated signaling transduction.^34^ On the other hand, in addition to LATS1/2, other kinases have been identified to account for the phosphorylation of YAP in response to various extracellular signals, for example, osmotic stress promotes Nemo-like kinase-dependent YAP phosphorylation at Ser128 and induces YAP activation.^35, 36^ Cellular energy stress induces AMP-activated protein kinase-mediated YAP Ser 94 phosphorylation and inhibition.^37, 38, 39^ In line with these studies, our data support that the phosphorylation of YAP by IKKβ and IKKε enhances the association between YAP and TEADs, which contributes to the target gene expression. Here, it is the first time to our knowledge that we report IKKs function as the upstream kinases of YAP through phosphorylation, which leads to YAP activation upon TNFα stimulation.

Two recent studies revealed there exists crosstalk between YAP/TEAD and other transcriptional factors including AP-1^40, 41^ or E2F1.^42^ YAP/TEAD form a complex with AP-1 or E2F1 that synergistically activates target genes directly involved in the control of S-phase entry and mitosis, indicating that YAP/TEAD probably acts as a transcriptional partner of other transcription factors complexes to accomplish fine-tuned regulation of the target gene expression under different stimulations. It has been reported that, p65, a key DNA-binding component of NF-κB, forms a complex with TEAD1 to regulate a subset of genes controlling the innate immune response.^29^ In agreement with these findings, we observed a strong association between YAP and p65 induced by TNFα treatment. Furthermore, YAP and p65 occupy at the same region on the promoters of detected genes and synergistically regulate expression of HK2, CCL2 and IL-6. Our findings further demonstrate that the co-operation of YAP/TEAD with different transcriptional factors in different cell contexts.

HK2 is a major enzyme of the HK family proteins that convert glucose to glucose-6-phosphate, controlling the first step of glycolysis. Increased glycolysis promotes cancer cell invasion by altering the pH of the tumor microenvironment and upregulating matrix metalloprotease activity and activating the intracellular signaling.^43, 44^ We showed that TNFα treatment elevated the cellular glycolysis of breast cancer cells and promoted cell migration, whereas HK2 knockdown mitigated migration induced by TNFα or macrophage CM, indicating glucose metabolism might be a key cellular event that links inflammation and migration. In addition, HK2 could be a potential therapeutic target for the treatment of metastatic breast cancer.

In conclusion, we reported that TNFα-IKK-YAP/TEAD/p65-HK2 signaling axis mediates TNFα- or macrophage-associated pro-migration of breast cancer cells. We further identified IKKβ/ε as new upstream kinases of YAP signaling, indicating both canonical and non-canonical NFκB pathways regulate YAP transactivation. YAP and p65 could synergistically regulate HK2 expression and promote cell migration. Hence, our results shed light on the novel mechanism of YAP signaling activation through non-cell-autonomous manner in the process of macrophage-associated breast cancer metastasis.

## Materials and methods

### Cell culture and plasmids

The 293T and MCF7 cell lines were maintained in high-glucose Dulbecco’s modified Eagle’s medium (Invitrogen, Waltham, MA, USA) supplemented with 10% fetal bovin serum (Gibco, Grand Island, NY, USA), 50 U/ml penicillin, 50 μg/ml streptomycin, in 5% CO_2_ atmosphere at 37 °C. Human THP-1 cells (from ATCC, Manassas, VA, USA) were cultured in RPMI-1640 medium with 10% fetal calf serum, 50 U/ml penicillin and 50 μg/ml streptomycin. Macrophages were prepared from THP-1 cells using 100 ng/ml PMA (Sigma-Aldrich, St Louis, MO, USA) treatment for 3 days.

Complementary DNA of YAP was subcloned into pQCXIH and pEGFP-C2 expression vector. Myc-p65 complementary DNA was subcloned into pQCXIH expression vector. Flag-IKKα and HA-IKKβ/ε were gifts from Dr Hongbin Shu.

### shRNA and siRNA

All shRNA were cloned into pLKO.1-puro vector. Targets sequences are as follows (all listed in the 5′–3′ direction):

YAP: GACATCTTCTGGTCAGAGA;

TEAD1/3/4: ATGATCAACTTCATCCACAAG;

HK2: GCAGAAGGTTGACCAGTATCT;

p65-1#: GCCTTAATAGTAGGGTAAGTT;

p65-2#: CGGATTGAGGAGAAACGTAAA.

TNFR1 and IKKs siRNAs were synthesized by GenePharma (Suzhou, China) based on the following target sequence:

TNFR1-1#: CAAAGGAACCTACTTGTACAA;

TNFR1-2#: CTCCAAATGCCGAAAGGAA;

IKKα: AGGTGGAAGTGGCCCTCAGTA;

IKKβ: ATTGCCTCTGCGCTTAGATAC;

IKKε: GGATCACCACGGAGAAGCCGG.

### Lentivirus

pLKO.1-shRNA plasmids or control vector were co-transfected with the lentivirus packaging plasmids pCMV-VSVG and pCMV-dR8.12 into 293T cells for virus production. Forty-eight hours after transfection, supernatant was filtered through a 0.45 μm filter, and used to infect cells. Seventy-two hours after infection, cells were selected with 5 μg/ml puromycin (Invitrogen) in culture medium.

### Regants and antibodies

Human recombinant TNFα protein was purchased from PeproTech (Rocky Hill, NJ, USA). Src-inhibitor saracatinib, JAK-inhibitor tofacitinib, mitogen-activated protein kinase-inhibitor SB203580, STAT3-inhibitor S3I-201, p65-inhibitor JSH-23, IKK-inhibitors TPCA-1 and IKK-16 were perchased from Selleckchem (Houston, TX, USA), JAK-inhibitor PDTC was perchased from Abcam (Cambridge, MA, USA). Antibody against HK2, phospho-IKKα/β (Ser176/180) and phospho-IKKε (Ser172) were purchased from Cell Signaling Techonology (Beverly, MA, USA), antibody against YAP was purchased from NOVUS Biologicals (Littleton, CO, USA), antibody against CD68 was pursed from Abcam, antibody against p65 were purchased from Santa Cruz Biotechnology (Santa Cruz, CA, USA). Antibodies against β-actin, GFP, Myc, Flag, HA and TEAD4 were purchased from Sigma-Aldrich. Antibody against TNFR1, IKKα, IKKβ and IKKε were purchased from Abclonal Technology (Wuhan, China). Antibody against pan-phosphorylated serine/threonine was purchased from BD Biosciences (San Jose, CA, USA).

### Western blot and immunoprecipitation

Western blot analyses were conducted as described previously.^45^ The cells were lysed by IP lysis buffer (50 mM Tris-HCl at pH 7.4, 150 mM NaCl, 1 mM EDTA, 1 mM EGTA, 5 mM Na_4_Ppi, 25 mM NaF, 1% Triton X−100, 1 M PMSF, protease inhibitor cocktail) on ice for 20 min, then were centrifuged at 12000 r.p.m. for 15 min, the supernatants were collected and incubated with IgG or specific antibody-conjugated beads for 3 h. Immunoprecipitates were washed for five times with IP wash buffer (50 mM Tris-HCl at pH 7.4, 150 mM NaCl, 1 mM EDTA, 1 mM EGTA, 5 mM Na_4_Ppi, 25 mM NaF, 0.5% Triton X−100) and centrifuged at 3000 r.p.m. for 3 min each time. Then the proteins were eluted by sodium dodecyl sulfate–polyacrylamide gel electrophoresis loading buffer and were detected by Western blot.

### RNA isolation and quantitative real-time PCR (RT-PCR) assay

Total RNA was isolated from cells or tissues using TRIzol reagent (Invitrogen), and cDNA was synthesized by reverse transcription using random primer followed by real-time PCR assays with gene-specific primers in the presence of 2 × Taq Master Mix (CWbiotech, Beijing, China). The relative abundance of mRNA was calculated by normalization to β-actin. Sequence of the qPCR primer pairs (all listed in the 5′–3′ direction) are as follows:

β-actin: F—CATCACCATCTTCCAGGAG;

R—AGGCTGTTGTCATACTTCTC;

CTGF: F—CAGCATGGACGTTCGTCTG;

R—AACCACGGTTTGGTCCTTGG;

CYR61: F—CTCGCCTTAGTCGTCACCC;

R—CGCCGAAGTTGCATTCCAG;

HK2: F—GAGCCACCACTCACCCTACT;

R—CCAGGCATTCGGCAATGTG;

YAP: F—TAGCCCTGCGTAGCCAGTTA;

R—TCATGCTTAGTCCACTGTCTGT;

CCL2: F—CAGCCAGATGCAATCAATGCC;

R—TGGAATCCTGAACCCACTTCT;

IL-6: F—ACTCACCTCTTCAGAACGAATTG;

R—CCATCTTTGGAAGGTTCAGGTTG;

PKM2: F—ATGTCGAAGCCCCATAGTGAA;

R—TGGGTGGTGAATCAATGTCCA;

LDHA: F—ATGGCAACTCTAAAGGATCAGC;

R—CCAACCCCAACAACTGTAATCT;

LDHB: F—TGGTATGGCGTGTGCTATCA;

R—TTGGCGGTCACAGAATAATCTTT;

GLUT4: F—TGGGCGGCATGATTTCCTC;

R—GCCAGGACATTGTTGACCAG;

PFKL: F—GCCAAAGTCTTCCTCATCTACG;

R—GTGCTGGACCAGGTTGTAGG.

### *In vitro* kinase assay

Overexpressed HA-IKKβ/ε in 293T cells was immunoprecipitated with HA antibody-conjugated beads (Sigma-Aldrich) for *in vitro* kinase assay. *In vitro* kinase assay was carried out in the presence of 100 μM ATP in 30 μl reaction buffer containing 100 mM Tris-HCl (pH 7.4), 10 mM MgCl2 and recombinant GST-fused YAP protein. After incubation at 30 °C for 30 min, the reaction was stopped by adding protein-loading buffer, and proteins were separated on sodium dodecyl sulfate–polyacrylamide gel electrophoresis gels for subsequent western blot.

### Transwell migration assay

To assess cell migration *in vitro*, transwell migration chambers (Milli Hang Culture 24-well PET, 8 μm, PIEP12R48, Merck & Millipore, Billerica, MA, USA) were used. MCF7 cells suspended in 100 μl serum-free medium were placed in the upper chamber and the lower chamber was filled with 700 μl complete culture medium. The plate was incubated at 37 °C in 5% CO2 for 24 h, followed by fixation with 4% formaldehyde. The upper chamber was gently wiped with a cotton swab to remove the non-migrated cells, and the migrated cells on the underside of the chamber were stained with 0.2% crystal violet. To evaluate the complete transmigration of the cells, the membrane was washed by dimethylsulfoxide and calculated at the absorbance of 570 nm.

### ChIP assay

ChIP was performed using the Simple ChIP Enzymatic Chromatin IP Kit (Magnetic Beads) from Cell Signaling Technology according to the manufacturer’s protocol. MCF7 cells (4 × 10^6^) were harvested for ChIP and formaldehyde cross-linked protein-DNA complexes were precipitated with antibodies against YAP (Cell Signaling Technology), TEAD4 (Sigma-Aldrich) and p65 (Santa Cruz Biotechnology). As negative control, normal mouse IgG and normal rabbit IgG (Merck & Millipore) were utilized. Purified DNA fragments were amplified by real-time PCR with the appropriate primers. Sequences of the real-time PCR primer pairs are as follows (all listed in the 5′–3′ direction):

HK2 primer: F—CGTGTAGGAGACGAGCGGTTC,

R—GGAGTTCCTCTGCCCCTTTC;

IL-6 primer: F—CCACCCTCACCCTCCAACAAA,

R—GAGCCTCAGACATCTCCAGTCCTAT;

CCL2 primer: F—CAGGCTTGTGCCGAGATGTT,

R—GGGAAGGTGAAGGGTATGAA.

### Immunofluorescence microscopy

MCF7 cells were cultured on coverslips. At moderate density, cells were treated with 10 μM TNFα or vehicle for 15 min, and then cells were fixed with 4% paraformaldehyde for 15 min and permeabilized in 0.2% Triton X-100 in phosphate-buffered saline for 10 min. Cells were then blocked with 2% bovine serum albumin in phosphate-buffered saline for 1 h and probed with antibody against YAP (Abclonal Technology) overnight at 4 °C. Nuclei were stained with 4,6-diamidino-2-phenylindole. MCF7 cells were visualized using ZEISS microscopy (Carl Zeiss, Jena, Germany). The images were processed using Image J software (National Institutes of Health, Bethesda, MD, USA). YAP status was evaluated as *N*>C (mainly in nuclei), *N*=C (both in cytoplasm and nuclei) and *N*<C (mainly in cytoplasm).

### Immunohistochemical staining

Immunohistochemical staining for HK2, YAP and CD68 was performed on 78 formalin-fixed and paraffin-embedded tissues using the PV9000 method. After de-paraffinization and rehydration, epitope retrieval was performed in citrate buffer (pH 6.0) for 2.5 min at 120 °C. After peroxidase blocking, the slides were incubated in 10% normal goat serum for 30 min at room temperature followed by the primary antibody (HK2: Cell Signaling Technology, 1:100; YAP: NOVUS Biologicals, 1:400, CD68: Abcam, 1:50) overnight at 4 °C. The slides were incubated in signal enhancer for 30 min followed by horseradish peroxidase-conjugated secondary antibodies (Zhongshan Technology, Beijing, China) for 30 min at room temperature. The slides were washed with phosphate-buffered saline, incubated in 3,3’-diaminobenzidine substrate (Zhongshan Technology) and counterstained with hematoxylin. Negative control is the primary antibody with 10% normal goat serum.

Immunohistochemistry staining was independently evaluated by two pathologists. For YAP and HK2 immunostaining, the percentage of tumor cells containing no staining (negative), weak staining (weak positive), moderate staining (positive), and strong staining (strong positive) was recorded for each specimen; for CD68 immunostaining, the macrophage infiltration status was evaluated as no (grade 1), weak (grade 2), moderate (grade 3), strong/robust infiltration (grade 4) for each specimen. The informed consent was obtained from all patients or their relatives and all human tissue samples were obtained and handled in accordance with an approved Institutional Review Board application (the Committee on Medical Ethics, the Institute of Biophysics of the Chinese Academy of Sciences (Beijing, China))

### Glucose consumption and lactate production measurements

MCF7 cells were seeded on 24-well plates. The cells were washed twice with culture medium, and fresh culture medium was added. Twelve hours later, the cell culture medium was collected and frozen at −80 °C, and the number of cells in each well was counted using a cell counter (Invitrogen). The glucose concentration in the collected cell culture medium were determined by using the glucose assay kit (Applygen, Beijing, China), the lactate concentration were determined by the lactate assay kit (Kinghawk, Beijing, China) according to the manufacturer’s protocol.

### Statistical analysis

The experiments were repeated at least three times. The results are expressed as the means±s.d. as indicated. Statistical analysis of the data was performed with a two-tailed Student's *t*-test, or two-way analysis of variance (GraphPad Prism 5, GraphPad Software, Inc., La Jolla, CA, USA). *P*<0.05 was considered to be significant.
