# Supplementary material for: TNFα-YAP/p65-HK2 axis mediates breast cancer cell migration
Source: Oncogenesis. 2017 Sep 25;6(9):e383–. doi: 10.1038/oncsis.2017.83 (PMC5623908; doi:10.1038/oncsis.2017.83)
Supplement: Supplementary Figures [file oncsis201783x1.pdf]

Supplementary Figures

Supplementary Figure 1

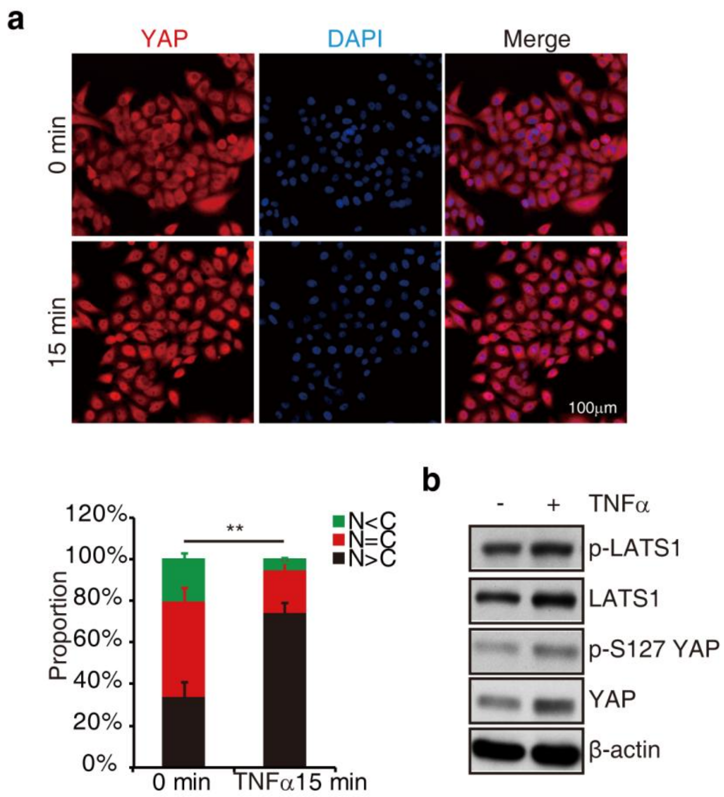

Supplementary Figure 2

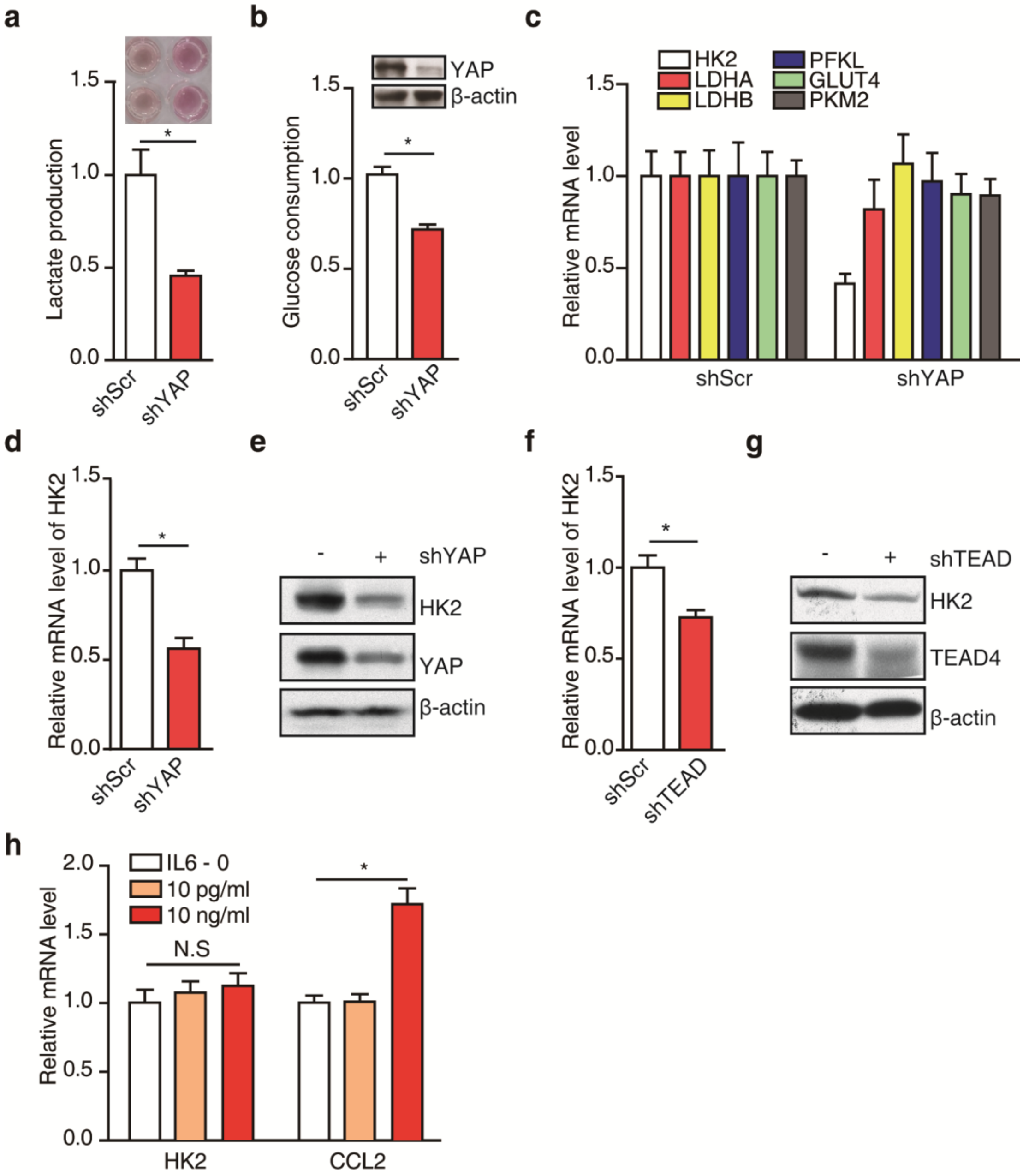

Supplementary Figure 3

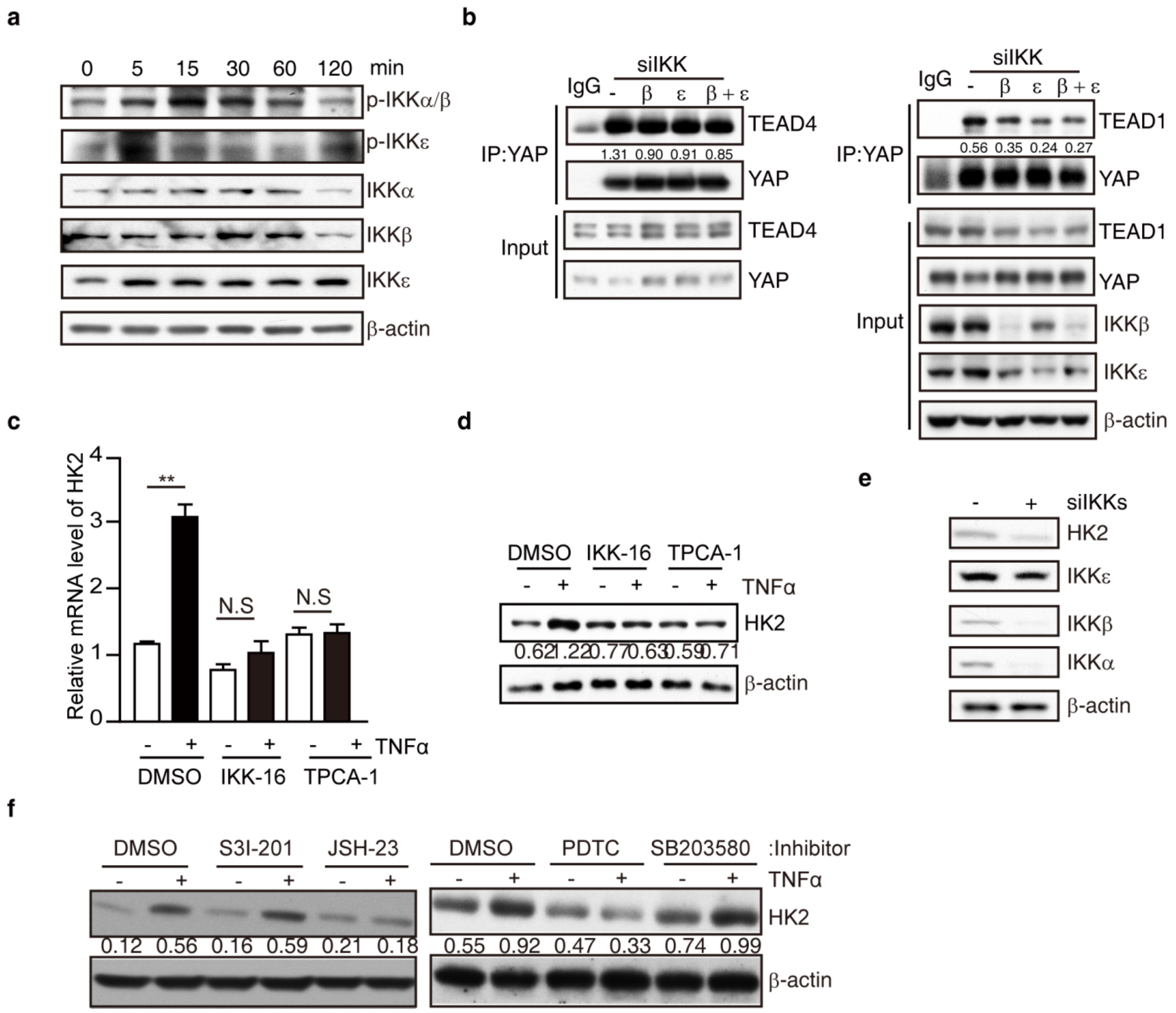

Supplementary Figure 1. TNFα induced an increased nuclear localization of YAP but independent on Ser127 phosphorylation.

(**a**) MCF7 cells at moderate density were treated with 10 μM TNFα for 15 min or not, YAP subcellular localization was examined by immunofluorescence. Typical images were shown (up) and the location was calculated from 10 fields (down). N>C (mainly in nucleus), N=C (both in cytoplasm and nucleus) and N<C (mainly in cytoplasm). The error bars represent the means ± SEM (**p< 0.01, n=10). (**b**) MCF7 cells were treated with 10 μM TNFα for 6h.The cell lysates were subjected to immunoblotting with indicated antibodies.

Supplementary Figure 2. YAP regulates the expression of HK2 and glycolysis in MCF7 cells.

(**a**) and (**b**) Control and shYAP MCF7 cells were assessed for lactate production (**a**), glucose consumption (**b**). (**c**) MCF7 cells stably expressing YAP-targeting or control shRNA were lysed for the detection of the mRNA levels of genes involved in glycolysis. (**d**) and (**e**) Control and shYAP-MCF7 cells were assessed for the expression of HK2 by real-time PCR (**d**) and Western blot (**e**). (**f**) and (**g**) Control and shTEAD MCF7 cells were analyzed for the expression of HK2 by real-time PCR (**f**) and Western blot (**g**). (**h**) MCF7 cells were treated with 10 ng/ml IL-6 for 12h and the mRNA levels of HK2 and CCL2 were measured by real-time PCR. The error bars represent the means ± SD (N.S: no significance; *p <0.05; n= 3; **p <0.01; n= 3; ***p< 0.001, n= 3).

Supplementary Figure 3. Inhibition of IKK/NF-κB pathway blocks the interaction between YAP and TEAD1/4 and reduces HK2 expression.

(**a**) MCF7 cells were treated with 10 μM TNFα for the indicated times. Cell lysates were subjected to immunoblotting with indicated antibodies. (**b**) MCF7 cells were transfected with siRNA against IKKβ, IKKε, or both respectively, 60h after transfection, cells were treated with 10 μM TNFα for 6h and then harvested and endogenous YAP was immunoprecipitated followed by immunoblotting with TEAD4

(left) or TEAD1 (right) antibody. (**c**) and (**d**) MCF7 cells were treated with TNFα combinated with 10 μM IKK-inhibitor TPCA-1 or IKK-16 for 12h, the expression of HK2 was analyzed by real-time PCR (**c**) and Western blot (**d**). (**e**) MCF7 cells were transfected with a mixture of siRNA against IKKα, IKKβ, and IKKε. 72h after transfection, cells were harvested and the lysates were subjected to immunoblotting with the indicated antibodies. (**f**) MCF7 cells were treated with 10 μM TNFα combinated with 10 μM STAT3-inbibitor S3I-201, 10 μM p65-inhibitor JSH (left) and PDTC or 10 μM MAPK-inhibitor SB203580 (right) for 24h, the cell lysates were used for the detection of HK2 protein expression *via* Western blot. The error bars represent the means ± SD (N.S: no significance; **p <0.01; n= 3).
